# Metagenomic insights into the microbial communities and functional traits of hot springs in Guizhou Province, China

**DOI:** 10.3389/fmicb.2025.1615879

**Published:** 2025-06-25

**Authors:** Feng Chen, Min Cheng, Dongyun Rong, Yanyan Wang, Rubing Liang, Muhammad Irfan, Yingqian Kang, Yu Cao

**Affiliations:** ^1^School of Public Health, The Key Laboratory of Environmental Pollution Monitoring and Disease Control, Ministry of Education & Guizhou Key Laboratory of Microbiome and Infectious Disease Prevention & Control & School of Basic Medical Science & Institution of One Health Research, Guizhou Medical University, Guiyang, China; ^2^Department of Nosocomial Infection Management, The Affiliated Hospital of Guizhou Medical University, Guiyang, China; ^3^State Key Laboratory of Microbial Metabolism, Joint International Research Laboratory of Metabolic & Developmental Sciences, School of Life Sciences and Biotechnology, Shanghai Jiao Tong University, Shanghai, China; ^4^ASRT, Inc., Atlanta, GA, United States; ^5^Department of Dermatology, The Affiliated Hospital of Guizhou Medical University, Guiyang, China

**Keywords:** thermophilic microorganism, extremophilic diversity, hot springs, microbial functional prediction, Guizhou Province, China

## Abstract

**Introduction:**

Hot springs were previously believed to be uninhabitable due to their hostile nature. However, recent studies have determined that hot springs not only have a rich microbiota but are also involved in various biogeochemical processes and possess unique characteristics that can be utilized for several biotechnological applications. This study aimed to determine the bacterial taxonomic diversity and functional profiles of 11 hot springs in the Guizhou Province, China.

**Methods:**

Illumina high-throughput sequencing was used to sequence the V3–V4 region of the 16S rRNA gene from microorganisms in samples collected from these hot springs. Software such as Mothur, the SILVA ribosomal RNA database, and Quantitative Insights into Microbial Ecology (QIIME) were utilized for taxonomic and operational taxonomic unit (OTU) analysis, while PICRUST2 was employed for functional predictions.

**Results:**

Guizhou Baili Rhododendron Hot Spring No.1 (BLDJA) had the highest diversity in terms of species richness, while Jianhe Hot Spring (YAS) had the lowest diversity. At the phylum level, the highest reported phyla included *Pseudomonadota, Bacillota, Nitrospirota, Bacteroidota*, and *Actinomycetota*, where *Pseudomonadota* had the highest abundance (92.094%) in Jianhe Hot Spring (YAS) and the lowest (41.238%) in Guizhou Baili Rhododendron Hot Spring No. 2 (BLDJB). *Bacillota* has the highest abundance (39.178%) in Guizhou Baili Rhododendron Hot Spring No. 2 (BLDJB) and the lowest (0.547%) in Jiutian Hot Spring (SNJT). The highest predicted functions were observed for amino acid metabolism, followed by carbohydrates. Predicted pathways for secondary metabolite and vitamin synthesis, along with stress-adaptation genes, underscore the biotechnological value of these habitats.

**Discussion:**

This study presents a preliminary survey of 11 hot springs in Guizhou Province, providing important insights into the origin and evolution of microorganisms. Furthermore, studying these microorganisms is crucial for understanding the adaptive mechanisms of life under extreme conditions, such as high temperatures, and for exploring the potential biotechnological applications of these microbes. An in-depth approach combining functional metagenomics and next-generation culturomics is required to fully understand the microbial flora and its potential biotechnological applications.

## 1 Introduction

Water sources with temperatures above 35°C are classified as hot springs (Chandrajith et al., 2013). As an analog of the primitive earth and an ancient model of life, hot springs have become an irreplaceable and attractive subject of study (Stan-Lotter and Fendrihan, 2012). Hot springs are a rich source of precious elements, various compounds, and diverse microbial communities. As representatives of extreme environments, hot springs have been shown to harbor a highly diverse and abundant microbial population, which still contains many uncultivated and unexplored microorganisms (Shu and Huang, 2022; Des Marais and Walter, 2019). These microbes have evolved a variety of metabolic systems to adapt to specific extreme environments (Nishiyama et al., 2018; Wani et al., 2022). They possess rich and unique genetic traits, metabolic products, and physiological adaptations that enable them not only to survive but also to thrive in such habitats. The most fascinating group of microbes is thermophiles, including bacteria and archaea, which can survive at high temperatures of 50–120°C. They inhabit high-temperature environments worldwide, including those caused by natural geothermal activity or anthropogenic conditions. Many of these habitats are geothermal springs, which are varied and occur on land, in shallow waters, or in the deep sea in the form of springs, geysers, or fumaroles (Burkhardt et al., 2024). Adaptation to hot spring environments requires genomic plasticity and metabolically flexible machinery in microorganisms to combat adverse conditions, making them good candidates for bioactive molecules for industrial and biotechnological applications (Strazzulli et al., 2017; DeCastro et al., 2016). Although hot-spring microbiology is well studied, comparable work on soil and marine thermophiles lags behind (Mahajan and Balachandran, 2017).

Terrestrial hot spring microbial populations have been a major focus of microbiological research. Hot springs around the world have been extensively studied, especially in Yellowstone National Park in the United States (Rowe et al., 2024; Saini et al., 2021), Japan (Asamatsu et al., 2021), India (Verma et al., 2022; Nagar et al., 2022), and Iceland (Podar et al., 2020). These sites differ in their physicochemical characteristics and microbial compositions.

China also has rich geothermal resources, especially in Yunnan (The Central People's Government of the People's Republic of China, 2024) and Guizhou provinces (The Central People's Government of the People's Republic of China, 2017) of western China, where hot springs are densely distributed. Geothermal regions such as Yunnan and Guizhou are located along the collision zone between the Indian Ocean Plate and the Asian Plate, sharing similar geological origins. Hot spring areas are primarily concentrated in western Yunnan and Guizhou, including Xifeng Hot Spring, Guizhou Baili Rhododendron Hot Spring, and Yuncong Duohua Hot Spring. However, the utilization of geothermal resources in Guizhou, China, remains limited to the primary stages of tourism, bathing, and electricity generation, and the deep exploration of the biological resources of hot springs remains underdeveloped.

In recent years, next-generation sequencing has emerged as a powerful tool. It is being increasingly used to study microbial populations in various environments and ecosystems, including oceans, soils, and geothermal reservoirs. Because hot springs are unique locations for thermophiles, the study of microbes in these systems is critical to our understanding of the diversity and evolution of life on Earth. This study aimed to explore the microbial diversity and composition in hot springs, focusing on thermophilic and thermotolerant bacteria with extraordinary functions and potential for industrial applications that inhabit hot springs in Guizhou Province, China. However, in recent years, these hot springs have suffered irreparable damage due to the commercialization and exploitation of geological tourism resources. Therefore, it is crucial to enhance the protection of hot springs to investigate their potential evolutionary mechanisms and industrial applications at an early stage.

## 2 Materials and methods

### 2.1 Sampling site and sample collection

**Figure 1:**
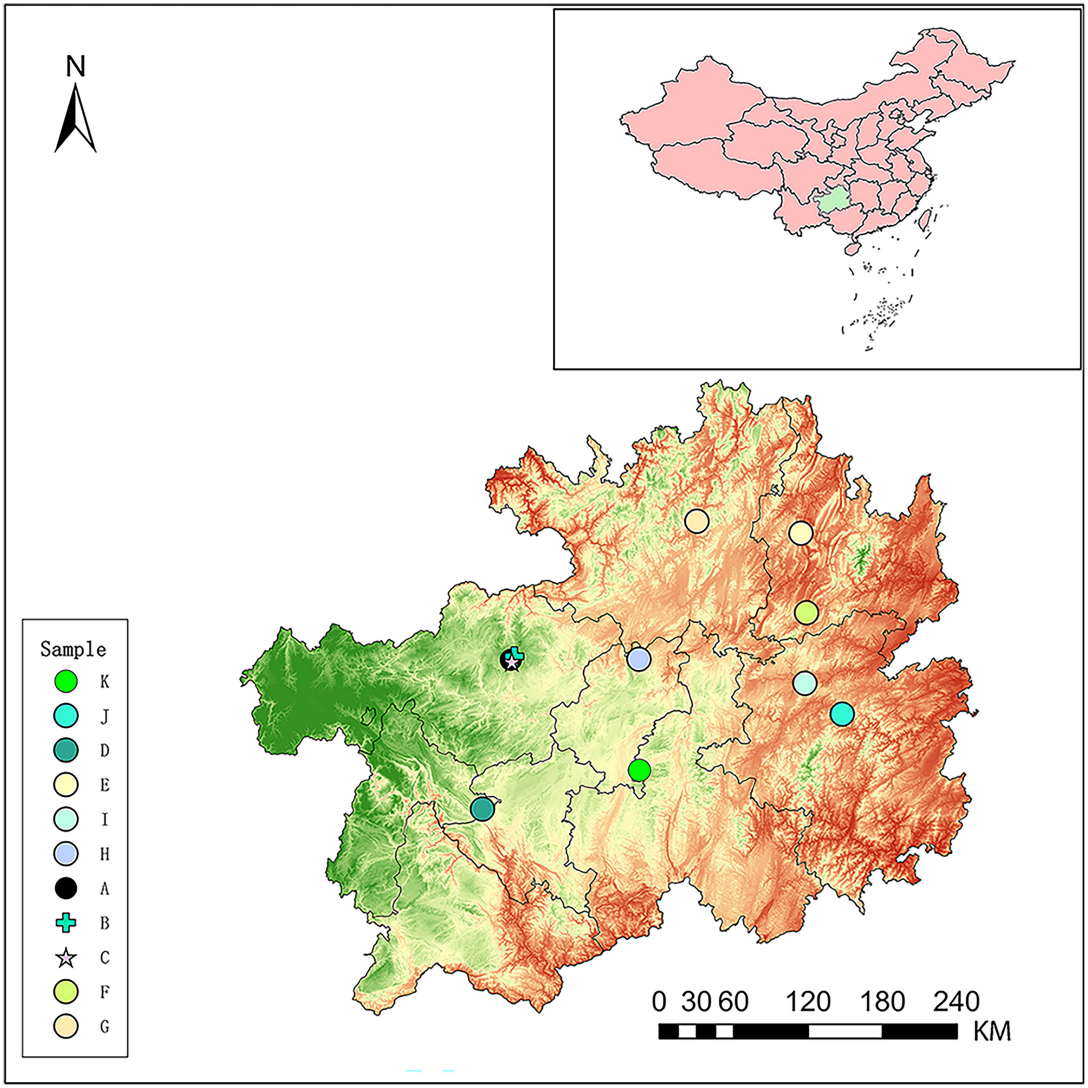
The illustration shows the locations of the sampling sites (hot springs) distributed throughout Guizhou Province, People's Republic of China. The sampling sites were noted as BLDJA (A), BLDJB (B), BLDJC (C), LZLJ (D), SNJT (E), SQ (F), SYSJ (G), XFLY (H), XFTM (I), YAS (J), and YCDH (K).

Eleven hot springs located in Guizhou province (Figure 1) were selected for the study, which are Baili Rhododendron Hot Spring No. 1 (BLDJA), Guizhou Baili Rhododendron Hot Spring No. 2 (BLDJB), Guizhou Baili Rhododendron Hot Spring No. 3 (BLDJC), Longjing Hot Spring (LZLJ), Jiutian Hot Spring (SNJT), Shiqian Hot Spring (SQ), Suiyang Crystal Hot Spring (SYSJ), Xifeng Hot Spring (XFLY), Xifeng Nanshan Tianmu Hot Spring (XFTM), Jianhe Hot Spring (YAS), and Yuncong Duohua Hot Spring (YCDH). The temperature and potential of hydrogen values were measured on-site using a handheld multi-parameter device, and the sampling site was positioned by a global positioning system along the latitude, as shown in Figure 1. Water samples were collected in triplicate from each hot spring using standard microbiological procedures. A total of 10 liters of hot spring water was filtered through a 0.22-μm membrane to concentrate the microorganisms onto the filter. Then, three filter membranes were collected and stored at −80°C for future use.

The temperature range of the Guizhou hot spring (11) was 37–72°C. The highest temperature recorded for Guizhou Baili Rhododendron Hot Spring No. 3 (BLDJC) is 72°C, followed by Guizhou Baili Rhododendron Hot Spring No. 2 (BLDJB) at 65°C and Yuncong Duohua Hot Spring (YCDH) at 60°C, respectively. The lowest recorded temperature was for Guizhou Baili Rhododendron Hot Spring No. 1 (BLDJA) at 37°C. The pH of the hot spring was slightly alkaline, with a pH of 8.93 measured at Xifeng Hot Spring (XFLY; Table 1).

**Table 1:** The study included the location details and physicochemical characteristics of 11 hot springs in Guizhou Province.

**Name**	**Sample**	**Longitude and Latitude**	**Temperature°C**	**Temperature (area) °C**	**pH**
BLDJA(A)	Guizhou Baili Rhododendron Hot Spring No. 1	N: 27°12′27″, E: 105°48′18″	37	24	7.19
BLDJB(B)	Guizhou Baili Rhododendron Hot Spring No. 2	N: 27°13′53″, E: 105°50′04″	65	20	7.24
BLDJC(C)	Guizhou Baili Rhododendron Hot Spring No. 3	N: 27°11′41″, E: 105°48′47″	72	22	8.35
LZLJ(D)	Longjing Hot Spring	N: 26°07′16″, E: 105°34′08″	43	28	8.76
SNJT(E)	Jiutian Hot Spring	N: 28°05′39″, E: 108°11′37″	45	30	8
SQ(F)	Shiqian Hot Spring	N: 27°30′53″, E: 108°13′19″	44	30	7.89
SYSJ(G)	Suiyang Crystal Hot Spring	N: 28°11′53″, E: 107°20′29″	51	30	8.39
XFLY(H)	Xifeng Hot Spring	N: 27°12′06″, E: 106°50′57″	47	30	8.93
XFTM(I)	Xifeng Nanshan Tianmu Hot Spring	N: 37°00′08″, E: 108°11′37″	45	29	7.97
YAS(J)	Jianhe Hot Spring	N: 26°46′15″, E: 108°29′24″	49	28	7.97
YCDH(K)	Yuncong Duohua Hot Spring	N: 26°23′37″, E: 106°50′22″	60	30	8.77

### 2.2 Amplicon sequencing of the 16S rRNA gene

Amplicon sequencing of the hot spring samples was outsourced by aseptically sending to the Beijing Genomic Institute, China. DNA was extracted from three enriched filter papers from each hot spring using the MagPure DNA extraction kit, according to the manufacturer's instructions. A total of 30 ng of high-quality metagenomic DNA was used for fusion-based PCR amplification. Polymerase Chain Reaction (PCR) parameters were set, and the resulting PCR products were purified using Agencourt AMPure XP reagent beads and dissolved in the elution buffer. Adapter ligation and indexing were performed to complete library construction. The size distribution and concentration of library fragments were assessed using the Agilent 2100 Bioanalyzer system, and libraries with appropriate insert sizes were selected for sequencing. Then, the circularization reaction was set up to obtain single-strand circular DNA. The final single-strand circularized library was amplified with phi29 and rolling circle amplification (RCA) to generate a DNA nanoball (DNB), which carries multiple copies of the initial single-stranded library molecule. The DNBs were loaded onto a patterned nanoarray, and paired-end reads of 300/250 base pairs were generated using the DNBSEQ-G400 platform (BGI-Shenzhen, China) to obtain the raw sequencing data.

#### 2.2.1 Processing of raw data

To generate high-quality reads and clean data (He et al., 2013), the obtained raw reads were first processed using cutadapt v.2.6 to remove the primers and connectors. Then, iTools fqcheck v.0.25 was used to discard reads with a Phred quality score below 20. Finally, readfq v1.0 was employed to filter out contaminated reads, ambiguous reads containing “N” bases, and low-complexity reads (e.g., sequences with 10 consecutive identical bases such as A, T, C, or G).

#### 2.2.2 Tags linkage and OTU clustering

First, FLASH (Fast Length Adjustment of Short Reads, v1.2.11; Magoč and Salzberg, 2011) was used to assemble the sequence, with a minimum match length of 15 bp and a mismatch rate of 0.1 in the overlapping region. Using overlapping regions, the paired-end reads obtained through double-end sequencing were assembled to generate tags corresponding to the highly variable region. Then, the spliced tags were clustered into operational taxonomic units (OTUs) using USEARCH (v7.0.1090; Edgar, 2013). Clustering was then performed using UPARSE at a 97% similarity threshold, and representative sequences for each OTU were identified. Finally, chimeras generated by PCR amplification were removed from the operational taxonomic unit (OTU) representative sequences using UCHIME (v4.2.40; Edgar et al., 2011) and the Chimera Database (version 20110519). The DADA2 (Divisive Amplicon Denoising Algorithm) method in the software QIIME2 (Caporaso et al., 2010) was used to denoise, and the filtered double-end sequences were imported using QIIME tools to obtain Amplicon Sequence Variants (ASVs) with 100% sequence similarity. The feature table was built using DADA2 with the QIIME dada2 denoise-paired command and then converted into a format that can be directly viewed by QIIME tools' export. The operational taxonomic unit (OTU) feature table was utilized for core-pan plots, diversity analysis, and principal coordinate analysis (PCoA). Species feature tables were used to perform species column graphs, graphical species composition maps, NMDS, LEfSe analysis, and KEGG functional analysis for functional prediction.

#### 2.2.3 OTU species annotation

After obtaining the OTU representative sequences, species annotation was performed using the RDP Classifier (v2.2; Wang et al., 2007) with a confidence threshold of 0.6. OTUs without annotation results were removed, as well as those annotated with taxa not classified as species in the Greengenes databases. The remaining OTUs were retained for subsequent analysis.

### 2.3 Bacterial community and functional prediction analysis

The Kruskal–Wallis non-parametric test was used to analyze bacterial community α-diversity (Schloss et al., 2009) indices using the Kruskal test package using R 3.2.1 across 11 hot springs, and box plots were generated based on the α-diversity indices. Bacterial β-diversity (Lozupone and Knight, 2005; Lozupone et al., 2011, 2007) across different hot springs was visualized using the ggplot package using R 3.4.1. Community bar charts were also created using R (v3.4.1) to illustrate the relative abundance and distribution of bacterial communities at the phylum and genus levels. Starting at the class level, species with a grouped average abundance below 0.5% and all unannotated at that classification were merged into the “Others” category. Meanwhile, the top 10 species were selected to show the mean relative abundance of each group and the significance of the difference test (^*^p ≦ 0.05, ^**^p ≦ 0.01, ^***^p ≦ 0.001). The metagenomic function was predicted by PICRUST2 (Douglas et al., 2019) based on 16S rRNA gene sequencing of 11 hot springs. Then, the three-level information on metabolic pathways was obtained from the KEGG database, and the abundance tables for each level were generated accordingly. An evolutionary clade plot was drawn using LEfSe (https://huttenhower.sph.harvard.edu/galaxy/).

## 3 Results and analysis

### 3.1 Community composition

**Figure 2:**
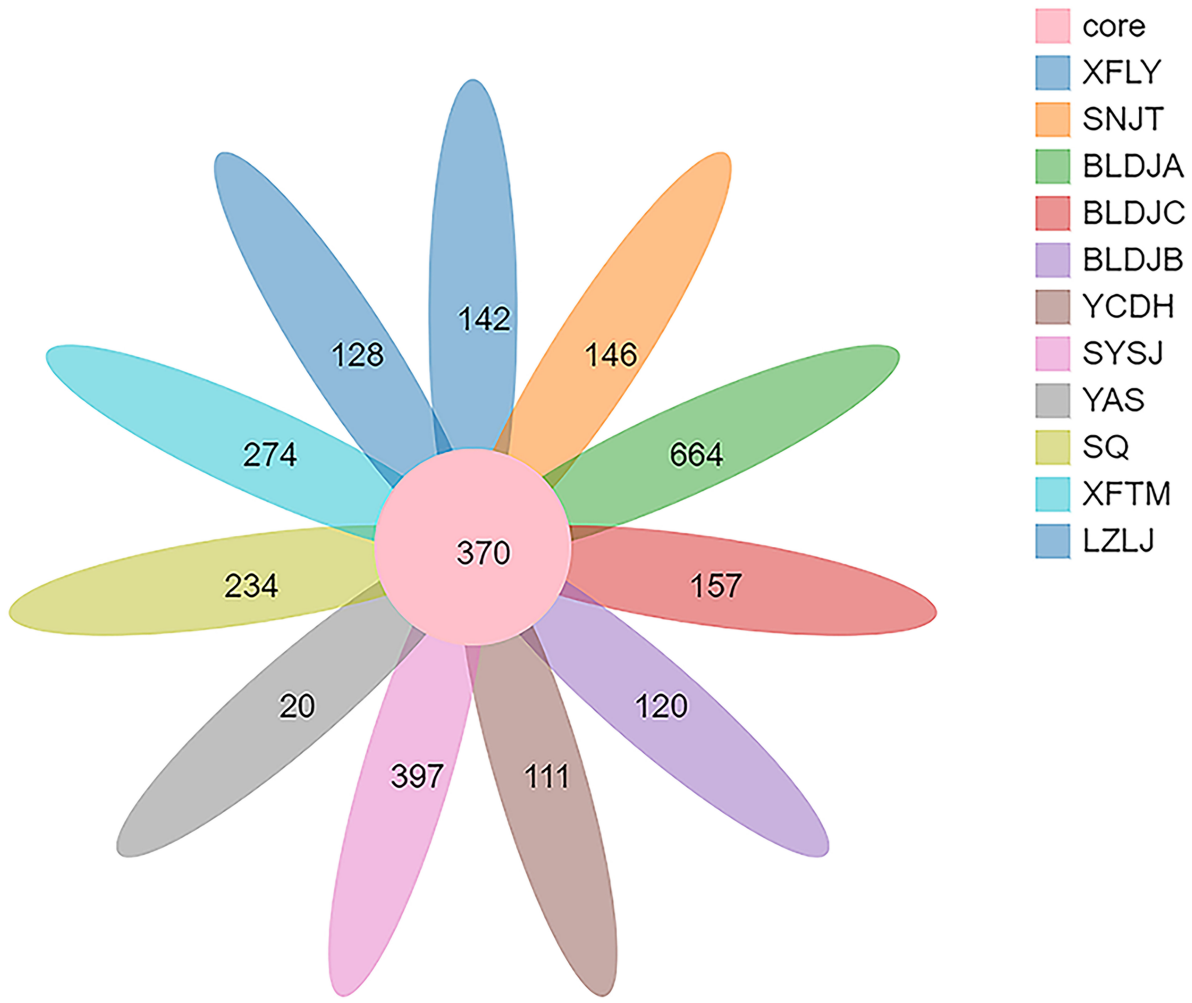
Core–Pan OUT richness and distribution of 11 hot springs in Guizhou.

High-throughput 16S rRNA sequencing of the bacterial populations in Guizhou hot springs yielded 174,034 high-quality reads, resulting in a total of 42,034 OTUs after filtering out low-quality sequences through optimization. The number of OTUs shared by all samples was 370, and the highest number of OTUs was 664 in Guizhou Baili Rhododendron Hot Spring No. 1 (BLDJA), followed by 397 OTUs in Suiyang Crystal Hot Spring (SYSJ). However, the lowest recorded number of OTUs for Jianhe Hot Spring (YAS) was only 20 OTUs (Figure 2). The results showed that hot springs with hostile environments are still rich in bacterial diversity; however, the bacterial community composition varies between hot springs in different regions in terms of richness and species distribution.

### 3.2 Alpha diversity analysis

**Figure 3:**
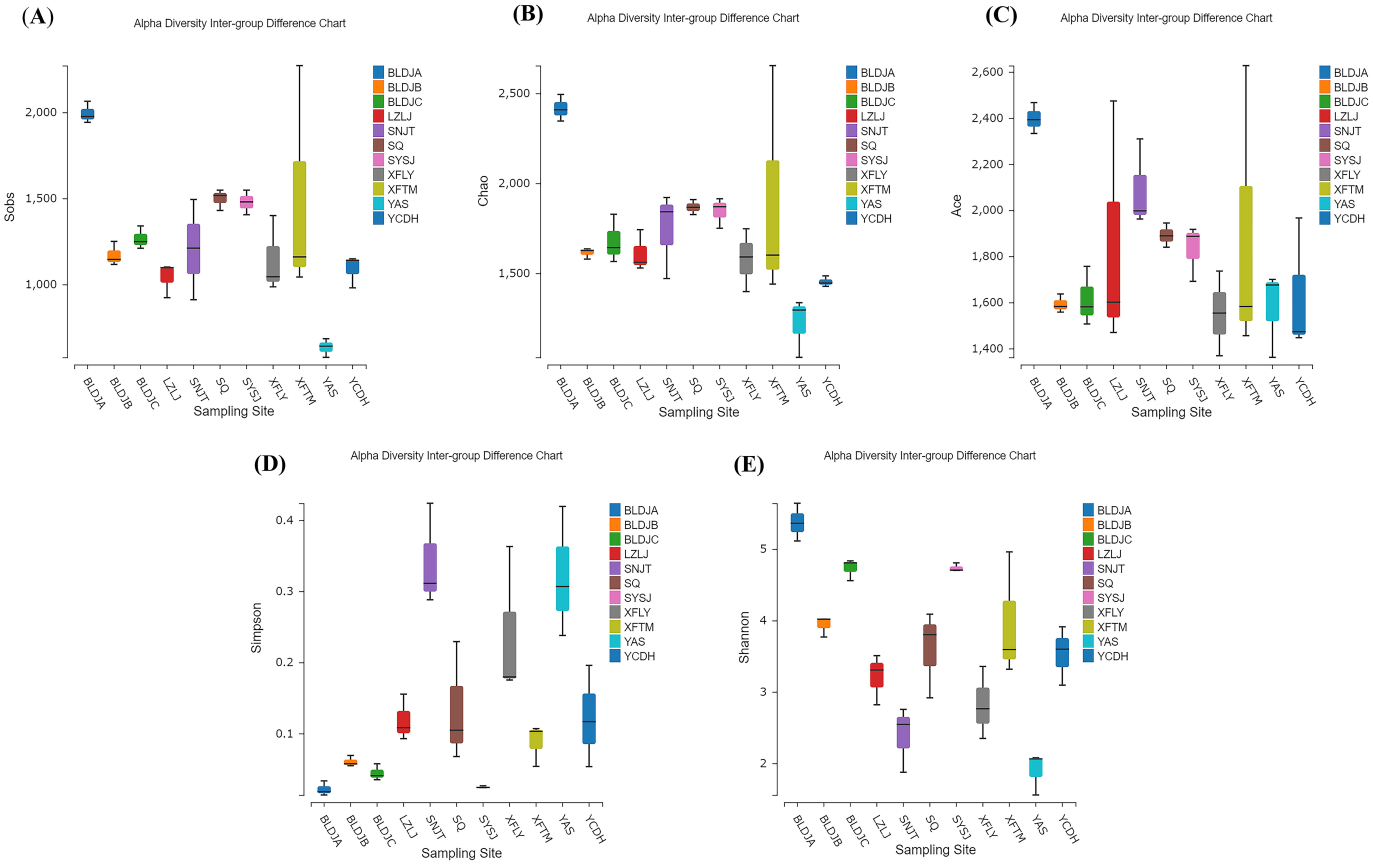
Different hot spring alpha diversity index box types in sobs **(A)**, chao **(B)**, ace **(C)**, simpson **(D)**, and shannon **(E)**.

As shown in Figure 3, there are significant differences in the alpha diversity index of 11 hot-source indigenous populations and among different types of native species. The above results indicate that Guizhou Baili Rhododendron Hot Spring No. 1 (BLDJA) has the highest richness, diversity, and evenness among different hot springs. Suiyang Crystal Hot Spring (YAS) exhibited the lowest richness and a low diversity index, indicating poor microbial diversity, which is consistent with the results shown in Figure 2. The distribution of each species was uneven, and the community structure observed was relatively simple.

### 3.3 Species composition

**Figure 4:**
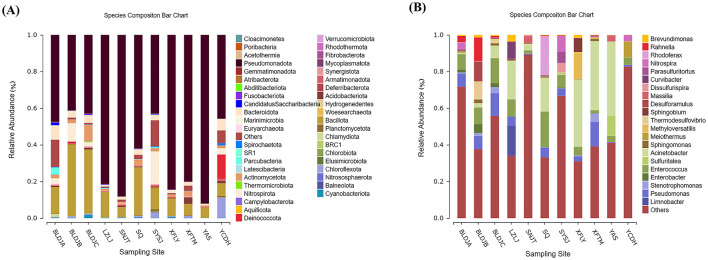
Distribution diagrams of different hot-source microbiological in phylum **(A)** and genus **(B)**.

At the phylum level, 16S rRNA gene sequences of 11 hot spring water bacteria were annotated to more than 40 phyla, such as *Pseudomonadota, Bacillota*, and *Nitrospirota* (Figure 4A), among which *Pseudomonadota* were the dominant bacteria in all hot springs, accounting for 41.24–92.09%. In addition, *Bacteroidota, Actinomycetota, Nitrospirota, Deinococcota, Chloroflexota*, and *Cyanobacteriota* were also detected in hot springs as the dominant group. Overall, the species had the same main groups but showed differences in relative abundance. In addition, *Cloacimonetes* (1%) and *Atribacterota* (1%) were only found in Guizhou Baili Rhododendron Hot Spring No. 1 (BLDJA), *Poribacteria* (1%) were found in Xifeng Nanshan Tianmu Hot Spring (XFTM), and *Acetothermia* (3%) was only found in Jianhe Hot Spring (YAS). There were two unique phylum groups in Suiyang Crystal Hot Spring (SYSJ), namely *Woesearchaeota* (0.001%) and *Nitrososphaerota* (1%).

At the genus level, *Meiothermus, Rhodococcus, Acinetobacter, Curvibacter, Rahnella, Pseudomonas*, and *Enterococcus* were the dominant bacterial genera in the different hot springs (Figure 4B). Species with an average cluster abundance of < 0.5% and all undetected species at that classification level have been merged into others. We have found that there are large numbers of unexplained species in each hot spring, accounting for 31.04–89.21%.

### 3.4 Analysis of microbial beta diversity

**Figure 5:**
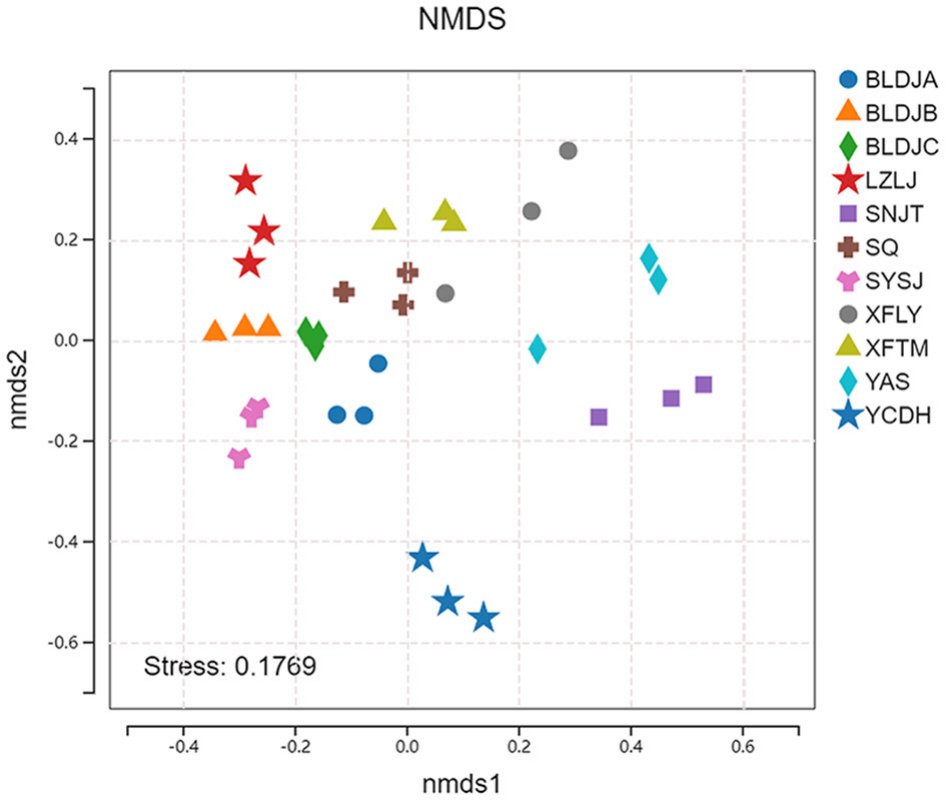
NMDS analysis of different springs' indigenous populations.

The non-metric multidimensional scaling (NMDS) results initially revealed differences in the beta diversity of native species across the various hot springs (Figure 5). The stress coefficient for the NMDS ordination analysis was 0.1769, which is below the threshold of 0.2, indicating that the ordination results are interpretable and hold meaningful ecological significance. A *p* < 0.05 and an R^2^ value approaching 1 indicate that the differences between groups are statistically significant and that the grouping is meaningful. Notably, the three sampling sites of Xifeng Hot Spring (XFLY) and Jianhe Hot Spring (YAS) were relatively scattered, indicating that the similarity of microbial community structure varied among the three samples from the same hot spring.

### 3.5 Differential microbiological analysis

**Figure 6:**
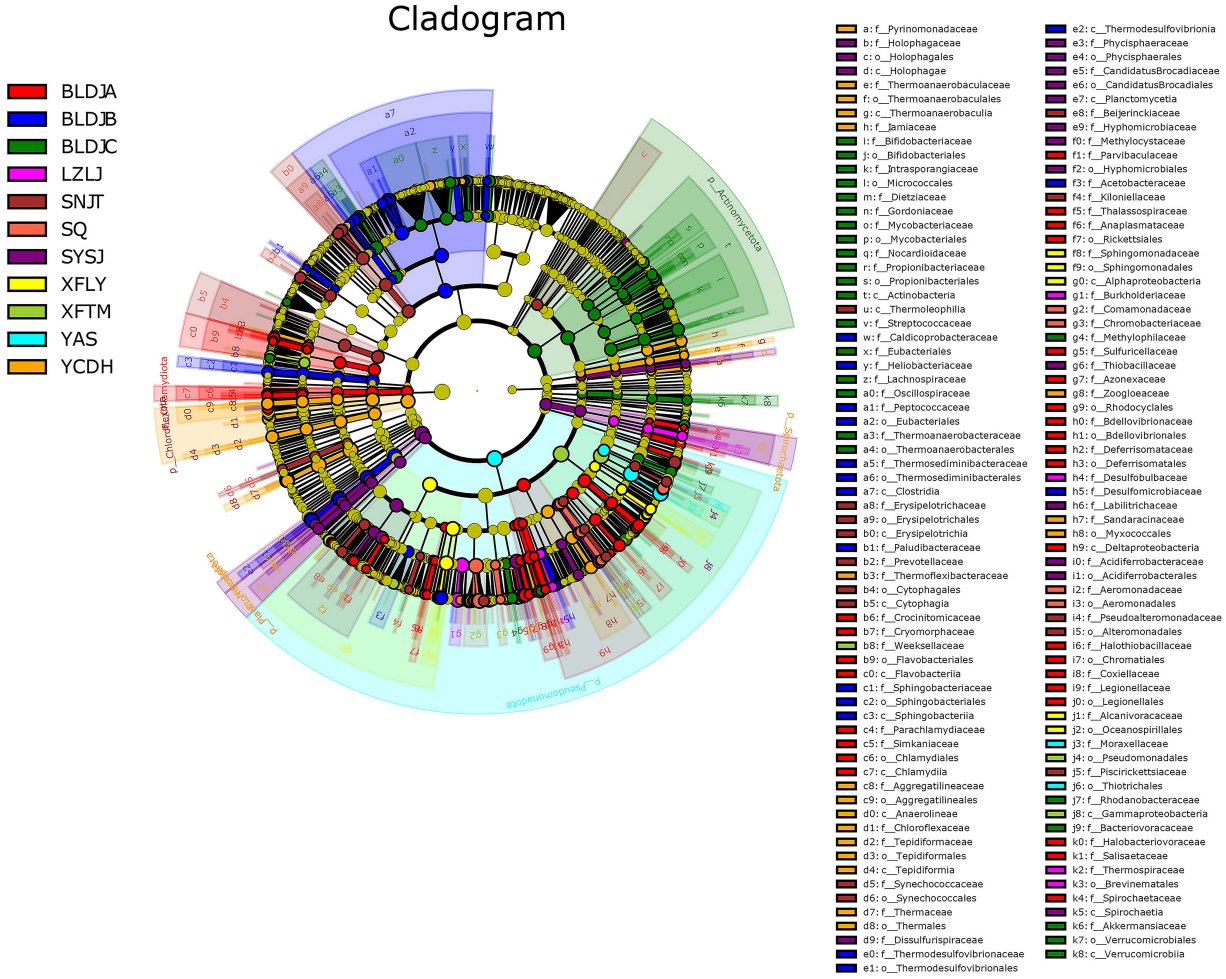
LEfSe analysis of different hot springs.

Linear discriminant analysis effect size (LEfSe) analysis was used to create a cladogram evolution clade map (Figure 6). The results showed that each of the 11 hot spring samples contained representative species with significant differences. The majority of microorganisms originated from the class level, with common *classes such as Pseudomonadota* and *Bacillota*, while a few microorganisms originated from the phylum level, including *Nitrospirota, Actinomycetota, Chloroflexota, and Deinococcota*. There were 63 species of microbiota in Guizhou Baili Rhododendron Hot Spring No. 1 (BLDJA). Mainly belongs to the *Chlamydiota* at the class level. Among these, there were three distinct evolutionary relationships with high contributions, including the transition from *Deltaproteobacteria* to *Bdellovibrio*, from *Gammaproteobacteria* to *Aquicella*, and from *Betaproteobacteria* to *Thiobacillus*. There were 36 characteristic species in Guizhou Baili Rhododendron Hot Spring No. 2 (BLDJB) with important indicative functions, which were different at the order level. The maximum evolutionary relationship of contribution was *between Nitrospirota* to *Thermodesulfovibrio* and *between Bacillota* to *Desulforamulus*. The characteristic species groups in Guizhou Baili Rhododendron Hot Spring No. 3 (BLDJC) differ at the class level, with 51 species, and primarily belong to the phylum *Actinomycetota*. For example, from *Actinomycetota* to *Bifidobacteriaceae, Bacillota* to *Lachnospiraceae*, and *Thermoanaerobacteraceae*. Longjing Hot Spring (LZLJ) has 13 characteristic species, all of which were classified at the order level, ranging from *Burkholderiaceae* to *Limnobacter* and *Comamonadaceae* to *Delftia*, including *Comamonas* and *Curvibacter*. In Jiutian Hot Spring (SNJT), 26 characteristic species played a vital indicator role, all of which were species at the class level or below. There was a complete evolutionary relationship from the family level to the species level, and the most different characteristic microorganism was *Chloroflexota*. Shiqian Hot Spring (SQ) has 12 characteristic species that play an important indicator role, and all the characteristic species were different at the family level. Suiyang Crystal Hot Spring (SYSJ) has 35 characteristic species with important indications, spanning from the class level, where there is a complete evolutionary relationship from the order level to the species level. Among them, four had the greatest contribution: *Alphaproteobacteria* to *Methylocystaceae, Betaproteobacteria* to *Parasulfuritortus, Gammaproteobacteria* to *Sulfuricaulis*, and *Nitrospirota* to *Dissulfurispira*. The 15 characteristic species that play an important indicator role in the Xifeng Hot Spring (XFLY) were all species at the genus level or below. From *Betaproteobacteria* to *Methyloversatilis* and *Hydrogenophaga, Alphaproteobacteria* to *Blastomonas* and *Caulobacter*. In Xifeng Nanshan Tianmu Hot Spring (XFTM), there were 12 characteristic species with important indicator functions, of which *Pseudomonadales* contributed the most, followed by *Xanthomonadales*, and then by *Stenotrophomonas*. Jianhe Hot Spring (YAS) has six characteristic species, all of which are classified at the phylum level or lower. Among them, the most divergent characteristic species group was *Pseudomonadota*, followed by *Betaproteobacteria*, then *Sulfuritalea*, and finally *Gammaproteobacteria*, with *Acinetobacter being the most closely related*. There were 39 characteristic species with important indicators in the Yuncong Duohua Hot Spring (YCDH), and the class level mainly belongs to *Spirochaetota, Planctomycetota*, and *Nitrospirota*. Three major evolutionary relationships contributing significantly are *Deinococcota* to *Meiothermus* and *Calidithermus*, followed by *Chloroflexota* to *Anaerolineae, Chloroflexus*, and *Tepidiforma*. Finally, *Pseudomonadota* to *Sandaracinus*.

According to the above results, the majority of bacteria in all hot springs primarily originate from *the Pseudomonadota* and *Bacillota domains*. This suggests that the microbial composition in different hot springs is roughly similar at the phylum level, but the proportions of various bacterial groups vary. Additionally, a small number of bacteria did not evolve from these two phyla, indicating that each hot spring has its own microbial evolutionary relationships. Compared with Shiqian Hot Spring (SQ), Xifeng Nanshan Tianmu Hot Spring (XFTM), and Jianhe Hot Spring (YAS), Guizhou Baili Rhododendron Hot Spring No. 1 (BLDJA), Guizhou Baili Rhododendron Hot Spring No. 3 (BLDJC), and Yuncong Duohua Hot Spring (YCDH) have more obvious differences in communities.

### 3.6 Comparison of the differences between key species

**Figure 7:**
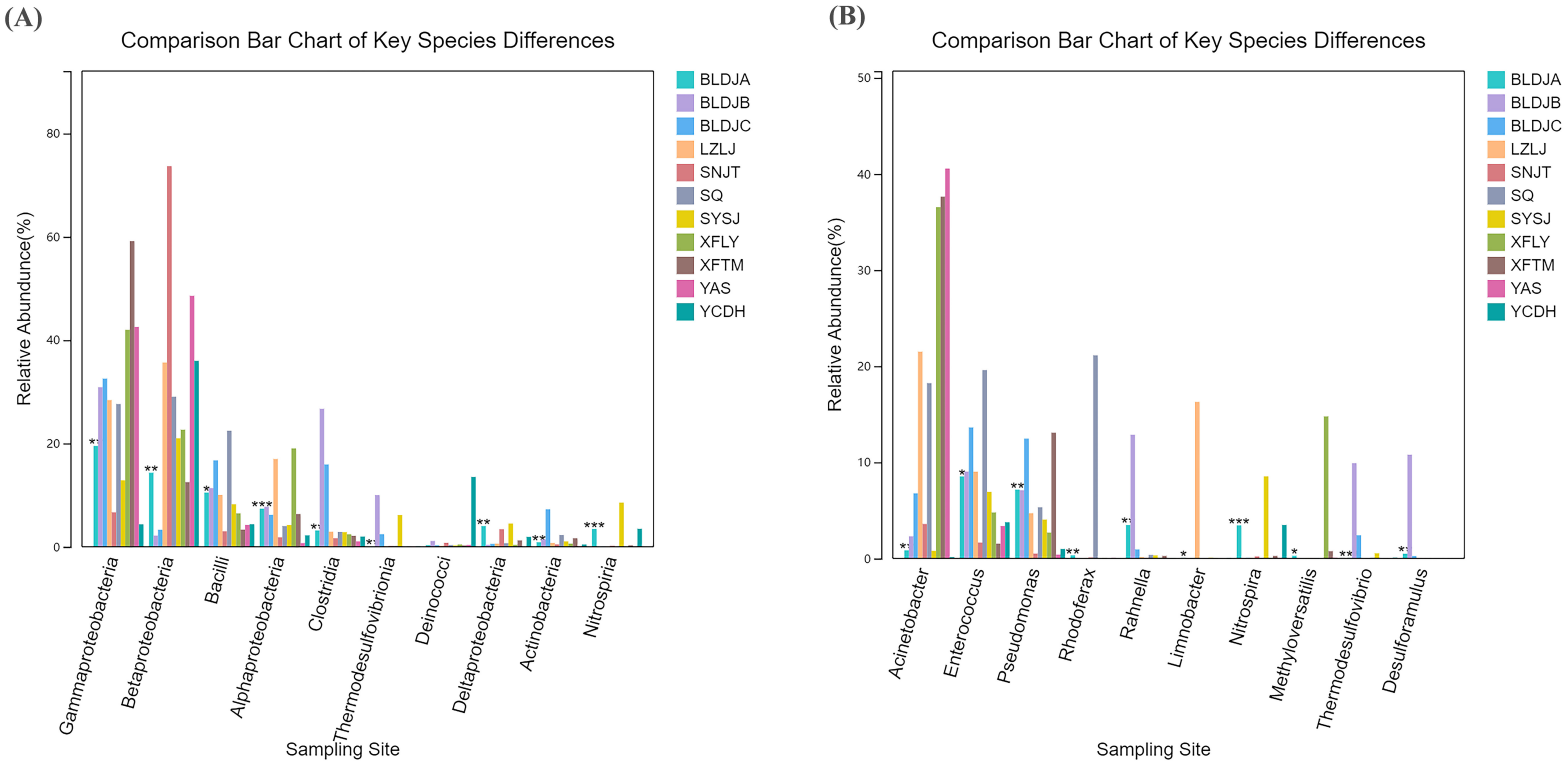
Differences between critical species in different hot springs in class **(A)** and genus **(B)**.

By analyzing the structure of microbial populations, information on the abundance and species composition of the eleven hot springs was obtained at different classification levels. After using linear discriminant analysis effect size (LEfSe) for differential species analysis, the specimens of the top 10 most abundant microorganism species were selected, and the relative abundance differences were compared at the species level. At the class level, except for *Deinococcota*, the relative abundance of the remaining nine microbial species varied across different hot springs (Figure 7A). Among these, *Nitrospirota* showed the largest difference in Guizhou Baili Rhododendron Hot Spring No. 1 (BLDJA; p < 0.001), containing 0.0351%, and the highest 0.1816% in Suiyang Crystal Hot Spring (SYSJ). The difference in *Cyanobacteriota* was minimal, with a *p*-value of < 0.05 and 0.0014% in Guizhou Baili Rhododendron Hot Spring No. 1 (BLDJA) and the highest content of 0.0176% in Guizhou Baili Rhododendron Hot Spring No. 3 (BLDJC).

At the genus level, the relative abundance of ten microorganisms varied in different hot springs (Figure 7B). Among these, *Nitrospira* showed the most significant difference, with a relative abundance of 0.0343% in Guizhou Baili Rhododendron Hot Spring No. 1 (BLDJA) and the highest abundance of 0.0854% in Suiyang Crystal Hot Spring (SYSJ), with a *p* < 0.001. The smallest difference was observed for *Enterococcus, Limnobacter*, and *Methyloversatilis*, with *p* < 0.05, in Guizhou Baili Rhododendron Hot Spring No. 1 (BLDJA). The *Enterococcus* presence was 0.0852% in Guizhou Baili Rhododendron Hot Spring No. 1 (BLDJA), and the highest abundance was 0.1960% in Shiqian Hot Spring (SQ). *Limnobacter* abundance was 0.0003% in Guizhou Baili Rhododendron Hot Spring No. 1 (BLDJA), and the highest abundance was 0.1629% in Longjing Hot Spring (LZLJ). The occurrence of *Methyloversatilis* Guizhou Baili Rhododendron Hot Spring No. 1 (BLDJA) was 0.0027%, and the highest presence in Xifeng Hot Spring (XFLY) was 0.1477%.

### 3.7 Functional diversity

**Figure 8:**
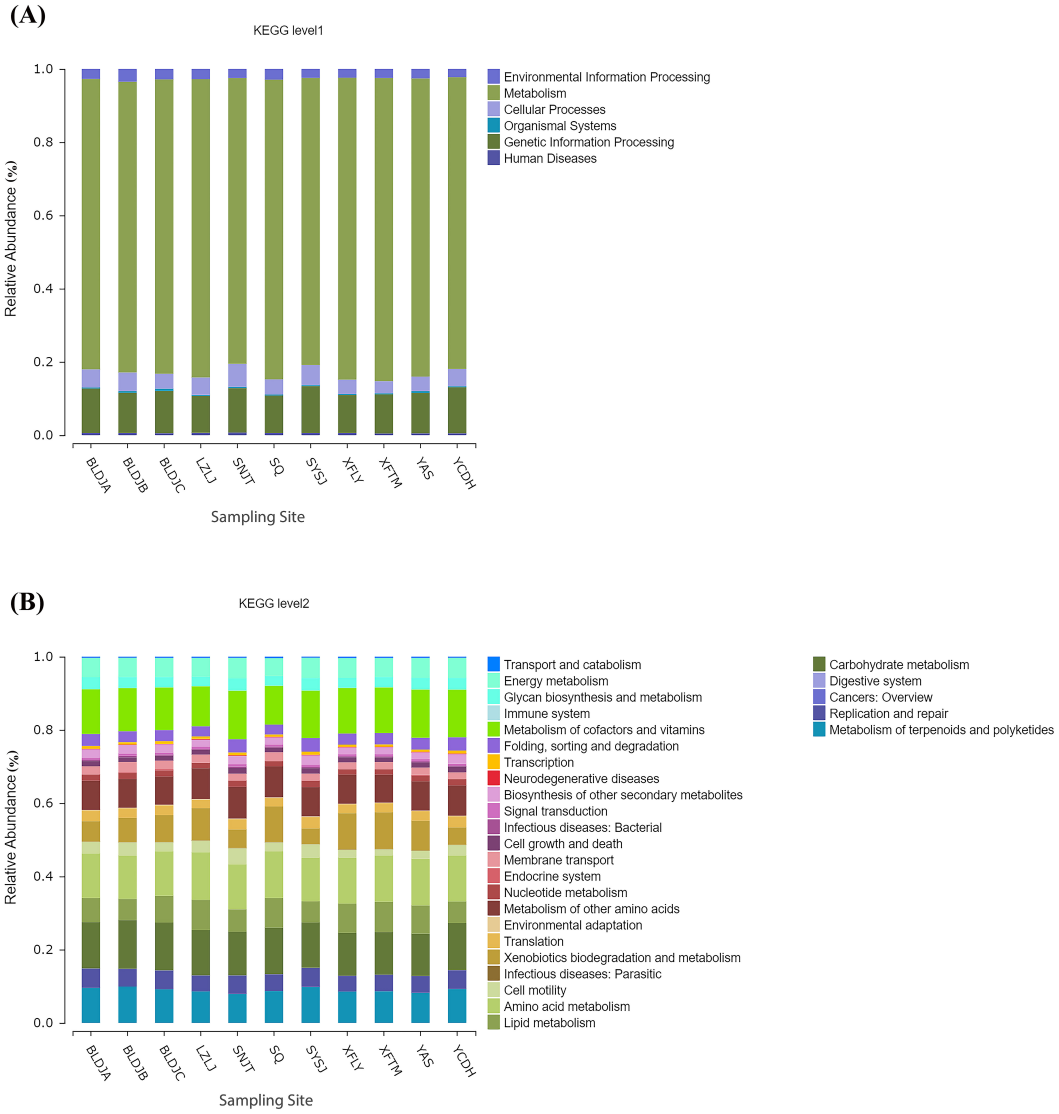
Comparison of KEGG level 1 **(A)** and level 2 **(B)** functions for different hot springs.

Obtain abundance predictions of bacterial community functions, such as clusters of orthologous groups of proteins (COG), metacyc, and Kyoto Encyclopedia of Genes and Genomes (KEGG) through Phylogenetic Investigation of Communities by Reconstruction of Unobserved States 2 (PICRUST2). The Kyoto Encyclopedia of Genes and Genomes (KEGG) function, known as KEGG Orthology (KO) Identity Document (ID), represents a specific functional gene and provides three levels of information on metabolic pathways based on the information in the Kyoto Encyclopedia of Genes and Genomes (KEGG) database. Each level was given an abundance table. The results showed that at level one (Figure 8A), all hot springs were metabolically very active, and a diverse range of biological functions were carried out, ranging from 78.02 to 82.79%. Additionally, the presence of related genes, such as those involved in genetic information processing and cellular functions, was detected. At level two (Figure 8B), carbohydrate metabolism, amino acid metabolism, the metabolism of cofactors and vitamins, and the metabolism of terpenoids and polyketides were the leading metabolites, with a small number of other secondary metabolites also being biosynthesized.

**Figure 9:**
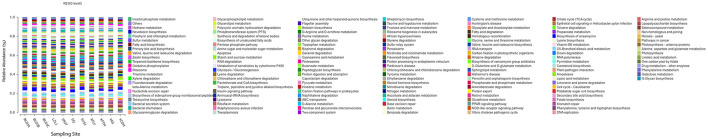
Comparison of KEGG level 3 functional categories for different hot springs.

In the Kyoto Encyclopedia of Genes and Genomes (KEGG) level 3 function prediction, we found that primary functions such as bacterial chemotaxis, base excision repair, and nucleotide excision repair can help microorganisms adapt to the environment and survive (Figure 9). To further understand the application of metabolites in different industries, we handpicked several important and abundant pathways in KEGG, as shown in Table 2.

**Table 2:** Potential for biotechnological applications.

**Major groups**	**Pathways**
Medical	Biosynthesis of ansamycins
	Biosynthesis of vancomycin group antibiotics
	Drug metabolism—other enzymes
	Flavonoid biosynthesis
	Novobiocin biosynthesis
	Penicillin and cephalosporin biosynthesis
	Streptomycin biosynthesis
Industrial	Betalain biosynthesis
	Carotenoid biosynthesis
	Terpenoid backbone biosynthesis
	Tetracycline biosynthesis
Environmental	Atrazine degradation
	Benzoate degradation
	Bisphenol degradation
	Dioxin degradation
	Limonene and pinene degradation
	Naphthalene degradation
	Polycyclic aromatic hydrocarbon degradation
	Toluene degradation
	Styrene degradation

## 4 Discussion

### 4.1 Microbial diversity

Guizhou province has 264 hot springs and is considered one of the regions with the richest hot spring resources in China (The Central People's Government of the People's Republic of China, 2017). These hot springs contain more than 30 kinds of trace elements, including 14 mineral elements for the human body—such as strontium, selenium, zinc, fluorine, iron, manganese, chromium, and vanadium—making them high-quality natural “baths” (Xiao-qing et al., 2014). These hot springs have high medical value and are effective against rheumatism, chronic digestive tract disease, chronic liver disease, biliary tract disease, diabetes, gout, cardiovascular disease, and hypertension (Chen, 2021). Guizhou Baili Rhododendron Hot Spring (BLDJA, BLDJB, and BLDJC) contains two valuable trace elements: fluorine, which, in appropriate amounts, aids in the absorption of calcium and phosphorus in the human body, and lithium, which helps regulate central nervous system activity and has soothing and calming effects. Lithium rarely accumulates in groundwater, so lithium-rich springs are uncommon. However, the lithium content in Guizhou Baili Rhododendron Hot Spring water is 1.42 mg/L, reaching a medically significant concentration. Shiqian Hot Spring (SQ) is a magnesium sulfate and calcium bicarbonate hot spring that contains a variety of trace elements (Luan, 2016). The metasilicic acid content ranges from 35.57 to 48.72 mg/L, and the strontium content ranges from 2.91 to 4.26 mg/L, qualifying it to be classified as strontium–metasilicic acid compound natural mineral drinking water. Moderate intake of strontium has been shown to help prevent hypertension and cardiovascular diseases (Yong-kang and Gan-lu, 2020). The chemical type of Suiyang Crystal Hot Spring (SYSJ) is a carbonated, high-strontium, carbon dioxide radon spring. This spring contains high levels of selenium, strontium, metasilicic acid, fluorine, lithium, and other elements beneficial to the human body. Among them, the Sr^2+^ content in the hot spring water ranges from 5.26 to 11.17 mg/L, and the H_2_SiO_3_ content ranges from 30.16 to 44.72 mg/L, which can be classified as a strontium-metasilicic acid compound natural drinking mineral water (Xiao-qing et al., 2014). Clinical trials have shown that Suiyang Crystal drinking water has a beneficial treatment effect on gastrointestinal diseases, cardiovascular disease, blood uric acid and gout, neurodermatitis, urinary stones, diabetes, cancer, and postoperative rehabilitation (Xin et al., 2015). There are various types of hot springs in Guizhou, but in terms of the breadth and depth of research, they are far less extensive than those in Yellowstone National Park. In the early stage, Guizhou hot springs focused more on geological background and geochemical research, serving as a basis for the development of geothermal resources. In recent years, the development and utilization of hot spring biological resources have begun to attract attention, highlighting the many unanswered questions about hot spring microbial communities that remain worth investigating.

The microbial diversity of seven urban hot springs in Guizhou province was analyzed using 16S rRNA gene amplification and high-throughput sequencing techniques.

The microbial communities of the 11 hot springs share similar predominant bacterial groups, with *Pseudomonadota* and *Bacillota* as the predominant bacteria and *Cyanobacteria* and *Actinobacteria* as secondary but notable groups. Wang and Pecoraro (2021) reported that *Pseudomonadota* dominated the microbial communities in the alkaline sediments and water of Julong High-Altitude Hot Springs, Tianchi Volcano, Northeast China. Among the hot spring samples, *Bacillota* was identified as the most diverse phylum. Similarly, Samarasinghe et al. (2021) found *Bacillota* to be the primary microbial population in Sri Lankan hot springs. Mashzhan et al. (2021) discovered that *Firmicutes* dominated the microbial community in the Zharkent Geothermal Hot Spring.

Sahay et al. (2017) studied the two hot springs of Manikaran and Yumthang Hot Springs in the Indian Himalayas, demonstrating that *Bacillota* is the dominant group. Furthermore, *Cloacimonetes, Poribacteria, Acetothermia, Balneolota, Woesearchaeota*, and *Nitrososphaerota* are found only in separate hot springs. Bacteria account for a large proportion of this study's hot-source microbiome (>99%) compared to the abundance of archaea (< 1%). In summary, while the relative abundance of bacterial taxa in hot spring microbial communities may vary significantly across different environments, the dominant groups tend to be similar.

The species richness and evenness of Guizhou Baili Rhododendron Hot Spring No. 1 (BLDJA) and Xifeng Nanshan Tianmu Hot Spring (XFTM) were relatively high in the 11 hot springs, with the highest diversity and even distribution in Guizhou Baili Rhododendron Hot Spring No. 1 (BLDJA), which may be related to the lower pH (7.19) and the appropriate temperature (37°C). Many factors affect the microbial community of hot springs (Chan et al., 2017; Mathur et al., 2007; Tobler and Benning, 2011), such as temperature (Wang et al., 2013; Guo et al., 2020; Kumar, 2023; Kumar et al., 2023a,b), pH (Pagaling et al., 2012; Power et al., 2018; Loskutova et al., 2024), geographical location (Stout et al., 2009), and a variety of nutrients with chemical components (Uribe-Lorío et al., 2019) in hot springs. The effects of different factors on microbial communities are varied. However, temperature and pH are the most critical among the factors affecting microbial communities in hot spring environments (Loskutova et al., 2024). Many researchers have researched different types of hot springs. Guo et al. (2020) found that the microbial communities of alkaline hot springs were primarily affected by pH and temperature. In contrast, the microbial communities of acidic mesothermal springs were mainly impacted by sulfate concentration. In addition, the study by Kumar et al. (2024) suggests that the correlation between bacterial diversity and pH is weak, whereas temperature plays a dominant role in shaping microbial community structure. Several studies have investigated the relationship between microbial diversity and temperature (Kumar, 2023; Kumar et al., 2023a,b). Overall, microbial diversity was negatively correlated with temperature, a key factor in controlling hot spring microbial diversity. The similarity of microbial community composition among hot springs decayed with increasing temperature differences (Miller et al., 2009; Purcell et al., 2007; Yasir et al., 2021; Jiang et al., 2024; Negi et al., 2024). This finding explains why BLDJA hot springs have the highest microbial diversity. In addition, temperature is closely linked to the heat-tolerance mechanisms of hot spring microorganisms. High-temperature stress induces oxidative stress, DNA damage (Habibi et al., 2022), and protein dysfunction (Sinetova and Los, 2016), thereby impairing the physiological functions of thermophilic microorganisms. Thermophilic microorganisms resist these damages through their cell membranes, proteins, nucleic acids, and mechanisms that mitigate oxidative stress, thereby surviving in high-temperature environments. The cell membrane serves as the first line of defense for thermophilic organisms adapting to high-temperature conditions. The cell membrane structure of thermophilic bacteria is special. With the increased environmental temperature, the ratio of saturated fatty acids and cyclized fatty acids increases, effectively enhancing the cell membrane's thermal stability and preventing it from liquefying under high-temperature conditions (Zili et al., 2015). High-temperature environments will also accelerate the generation of reactive oxygen species in cells, and excessive reactive oxygen species will cause damage to proteins and DNA (Liu et al., 2020). To mitigate this oxidative damage, hot spring microorganisms stimulate the activity of antioxidant enzymes, such as superoxide dismutase (Barcyte et al., 2020) and catalase (Steimbrüch et al., 2022). Additionally, prolonged exposure to high-temperature environments may trigger genomic instability. Thermophilic microorganisms can produce heat-stable enzymes that maintain catalytic activity at high temperatures, ensuring normal metabolic processes within cells (Averhoff and Müller, 2010). Extreme thermophilic proteins exhibit enhanced DNA stability, protecting DNA molecules from degradation and damage caused by heat at high temperatures. For example, *Taq* polymerase retains its function at high temperatures, which is crucial for DNA replication (Stetter, 1999).

At the genus level, the dominant bacteria in Longjing Hot Spring (LZLJ), Jiutian Hot Spring (YAS), Xifeng Hot Spring (XFLY), Xifeng Nanshan Tianmu Hot Spring (XFTM), and Jianhe Hot Spring (YAS) are *Acinetobacter*, which are widely distributed in nature and are mainly found in water and soil. The tolerant pH range is 3.0–9.0 (optimal pH range 6.0–7.0). *Acinetobacter* is an important degrader of petroleum hydrocarbons, playing a crucial role in mitigating the biological toxicity of these compounds, thereby facilitating their use in petroleum hydrocarbon restoration engineering (Yu-hua et al., 2016). The dominant bacterium of Guizhou Baili Rhododendron Hot Spring 2 (BLDJB) and Guizhou Baili Rhododendron Hot Spring No. 3 (BLDJC) is *Thermodesulfitimonas*, which also shows potential applications in the field of microbial electrosynthesis. *Thermodesulfitimonas* was proposed as one of the likely major producers in electrochemically active biofilms of a novel Knallgas bacterium, a technique that combines microbial metabolism with electrochemical processes for carbon dioxide conversion and fixation (Reiner et al., 2020). The dominant bacteria in Suiyang Crystal Hot Spring (SYSJ) is *Nitrospira*, which plays an important role in the nitrogen cycle. They can oxidize nitrite to nitrate, a process crucial for maintaining the health of aquatic ecosystems (van Kessel et al., 2015). *Nitrospira* is the primary nitrite-oxidizing bacterium in sewage treatment, closely related to the ammonia nitrogen, nitrate, and nitrite cycle system, and is crucial for aquatic plants. In addition, some bacteria of the genus *Nitrospira* possess the full ammonia oxidation capacity (comammox), which enables them to directly oxidize ammonia to nitrate and have significant ecological and environmental implications in the nitrogen cycle (Vijayan et al., 2021). The dominant bacteria in Yuncong Duohua Hot Spring (YCDH) were *Meiothermus*, and they grow optimally at 50–65°C and pH 7–8.5. Some strains of the genus *Meiothermus* exhibit specific functions. For example, *Meiothermus ruber* contains genes involved in the TPS/TPP trehalose synthesis pathway, which have been cloned, expressed, and functionally characterized (Zhu et al., 2010). *Meiothermus* sp. *sk3-2* can convert maltose to trehalose, potentially serving as an alternative source for trehalose production (Goh et al., 2011).

### 4.2 Functional prediction diversity

Hot springs are considered valuable sources of bioactive compounds with significant potential for biotechnological applications in various fields. Many enzymes found in thermophilic microorganisms resist high temperatures, organic solvents, and decontaminants, providing unparalleled advantages in biometallurgy, wastewater treatment, food production, and papermaking (Cai-hong and Ye-jun, 2019). These microorganisms can produce valuable biotechnological products, such as antibiotics (Sahm et al., 2013), bioethanol (Bowen De León et al., 2013), and thermostable enzymes (Urbieta et al., 2015; Elleuche and Antranikian, 2013; Sharma et al., 2013). Exploring new sources of thermophilic microorganisms can enhance their research and utilization in various fields.

#### 4.2.1 Prediction of major adaptation mechanisms

In the Kyoto Encyclopedia of Genes and Genomes (KEGG) level 3 function prediction, we found that primary functions, such as bacterial chemotaxis, base excision repair, and nucleotide excision repair, enable microorganisms to adapt to their environment and survive. Maintaining genome stability and the integrity of genetic information is essential for the accurate transmission of the genetic code. However, during DNA replication, it is constantly threatened by DNA-damaging reagents from within bacteria or in the environment, which produce multiple damages (Chatterjee and Walker, 2017) through oxidation, alkylation, deamination, or hydrolysis. If these DNA lesions are not properly repaired, the important cellular processes will be compromised, threatening the survival of the organism (Chatterjee and Walker, 2017; Terabayashi and Hanada, 2018; Kiwerska and Szyfter, 2019). Therefore, cells must repair these damages in a timely manner. In response to the potential harm caused by DNA damage, organisms have evolved a series of complete repair pathways to monitor and repair DNA damage. The most common mechanisms of DNA damage repair can be divided into five categories: base excision repair, nucleotide excision repair, mismatch repair, homologous recombination, and non-homologous end joining (Kim et al., 2018; Spampinato, 2017). Cells typically select one or more repair pathways to complete damage repair, depending on the type of damage, the cell cycle, and the biological species. Among them, base excision repair is a common class of DNA damage repair pathway in all organisms, which is often used to repair multiple DNA damages (Wallace, 2014; Dizdaroglu et al., 2017), including alkylation, deamination, and oxidation, and consists of multiple enzymes. Nucleotide excision repair is a repair mechanism that recognizes broad-spectrum DNA lesions and is particularly important for the repair of certain broad lesions, such as crosslinks, large adducts, and multibase lesions (Kemp, 2019).

Given the heterogeneous and complex natural environment, bacteria have evolved multiple patterns of chemotaxis behavior to accommodate changes in their surrounding microenvironment (Adler, 1969; Sampedro et al., 2015). Bacteria often actively utilize their movement ability to respond to the concentration gradient of chemicals in the environment, transforming their random movement into biased movement. This behavior is known as “chemotaxis” (Adler, 1969), a fundamental attribute of bacterial adaptation to their environment. The influence of bacterial chemotaxis on microbial communities is primarily reflected in the regulation of microbial community structure and the promotion of interactions with the environment. Motile bacteria play a very important role in nature. They can promote material metabolism, drug delivery, and nutrient circulation through chemotaxis (Karmakar, 2021), such as forming a symbiont with other organisms or facilitating directional movement and migration of non-motile bacteria, known as hitchhiking. This form of movement can enable non-motile bacteria in the community to move and migrate, driven by motile bacteria, thus affecting the microbial composition and metabolism of substances within the community (Fukui et al., 1999). This movement mechanism enables bacteria to find a suitable environment for survival, regulate temperature, maintain pH balance, and control nutrient uptake, thereby avoiding harmful substances and escaping adverse environments. It can also promote the circulation, transformation, and distribution of substances and is significant in regulating the structural diversity of microbial communities (Xiao-yan et al., 2019).

#### 4.2.2 Biotechnological potential of the hot spring microbes

In 1831, the German Wachenreder was the first to isolate from the carrot root a carbohydrate pigment named “carotene” (Yong-hua and Shi-zhong, 2000). With the development and progress of society, domestic, and foreign scholars have continuously discovered and separated a series of other natural pigments through various technical methods, collectively known as “carotenoids” (Ke and Na, 2010). Natural pigments are primarily derived from plants and microorganisms, while plant-derived pigments have a relatively long growth cycle, which limits their large-scale applications. Microorganisms have broader application prospects than plant-source pigments due to their rapid growth, low nutritional requirements, minimal environmental impact, and low production costs. Several disadvantages of synthetic pigments have contributed to an increased demand for natural, organic, and eco-friendly pigments (Manikprabhu and Lingappa, 2013).

Carotenoids are a group of lipid-soluble pigments, also known as tetraterpenes, composed of eight 5-carbon isoprenoid molecules (Yong-hua and Shi-zhong, 2000), widely distributed in plants and microorganisms (Phadwal, 2005). Carotenoids are primarily produced by a wide range of phototrophic and non-phototrophic organisms, including bacteria, algae, fungi, and plants. Some bacteria and fungi, as non-photosynthetic species, also produce carotenoids to resist photooxidation, allowing them to survive in sufficient light and air. Carotene-producing bacteria encompass a diverse range of species, and all C45 and C50 carotenoids reported to date are synthesized by bacteria, with 107 bacterial species currently known to synthesize 307 carotenoids (Yabuzaki, 2017). The radiation energy captured and reflected by natural pigments serves a variety of biological functions, including harnessing solar energy for metabolism and shielding organisms from radiation damage.

Microbial pigments also exhibit potential biological activities, including antibacterial, anticancer, radiation resistance, and antioxidant properties, which promote the exploration of microbial pigments. Carotenoids have remarkable health benefits and play a significant role in regulating the body's immune function. In the early 1980s, carotenoids were discovered in *Seifter* and found to potentially enhance immune activity (Jun-hui, 2012). The presence of a certain number of highly active free radicals in the body can damage the cell membranes of normal cells, triggering a series of chain reactions that lead to a sharp decline in cell function and rapid aging of the body. Carotenoids, especially beta-carotene, have a notable effect on eliminating free radicals and delaying the aging process.

Additionally, they have positive preventive and therapeutic effects on coronary heart disease, thrombosis, and other diseases (Ling, 2008). It also plays a role in cancer prevention and anticancer effects (Giovannucci et al., 1995). The cancer prevention and treatment process not only inhibits the occurrence of a variety of cancers but also reduces the cancer mortality rate by 20–30% (Lan and Hao-ming, 1998). Carotenoids have been widely used in the healthcare, food, and cosmetics production industries for their antioxidant and anti-tumor properties (Borowitzka, 2013).

In 1918, betalain was isolated from red beetroot. It is a water-soluble, nitrogen-containing pigment belonging to quinone derivative pigment, soluble in water and polar solvents but insoluble in the majority of organic solvents (such as ethanol, methanol, acetone, ethyl acetate, and so on; Yan et al., 2021). Similar to many natural pigments, betalain has unstable structures and can be easily affected by temperature, light, pH, metal ions, and other environmental factors. Red beetroot has long been the sole source of betalain extraction. However, high levels of nitrates and earthy flavors in beetroot extract limit the development and utilization of beetroot pigments (Rodriguez-Amaya, 2019). Therefore, it is important to find other sources of betalain in nature. Currently, 75 types of betalain structures have been identified from 17 different plants (Khan and Giridhar, 2015). Betalain has high physiological activity, with antibacterial (Spórna-Kucab et al., 2018) and anti-fatigue (Rodriguez-Amaya, 2019) effects. As research continues to deepen, it has been found that betalain has an impact on cancer (Mancini et al., 2021; Lechner and Stoner, 2019), preventing coronary heart disease (Haswell et al., 2021), and lowering blood lipids (Rahimi et al., 2019), indicating that there is significant application potential in the field of betalain drug development. Moreover, due to the efficient coloring properties of betalain, it can also be used in the production of cosmetics. Betalain has a natural hue, can prevent UV rays, eliminate facial free radicals, and maintain facial moisture. It is easy to apply, wash off, and biodegradable, making it suitable for the development of blush products (Ping and Ming-zhe, 2021).

Microbial drugs play an indispensable role in the field of antimicrobial therapy. Currently, 70% of clinical antibiotics are derived from microbial natural products or their derivatives, which has saved hundreds of millions of lives. In addition to the two main groups of *Actinomycetes* and fungi, microorganisms that produce drugs also include *Pseudomonas, Bacillus, Myxobacteria, Cyanobacteria*, and others, as well as microbial communities from extreme environments. Among the 293 clinical drugs derived from microorganisms, 143 are from *Actinomycetes*, accounting for 49%; 121 are from fungi, accounting for 41%; and 29 are from bacteria, accounting for 10%. Among the 105 microbial drugs derived directly from natural products, 69 are from *Actinomycetes*, accounting for 66%; 17 are from fungi, accounting for 16%; and 19 are from bacteria, accounting for 18% (Jian-hua et al., 2021).

Antibiotics are a type of secondary metabolite or their artificial derivatives produced during the life activities of microorganisms or other biological organisms, which can inhibit or affect the life activities of other organisms at low concentrations (Qin-xiang, 2008). Antibiotics primarily act in the treatment of bacterial diseases. In general, antibiotics are drugs used to treat bacterial infections and infections caused by pathogenic microorganisms. Penicillin is the first known antibiotic found in the history of human development, but penicillin does not inhibit and kill all pathogens. Penicillin is mainly effective against Gram-positive bacteria (Qin-xiang, 2008). The mechanism of action of penicillin primarily targets the synthesis of cell walls in Gram-positive bacteria. In 1943, soil microbiologist Selman Waxman isolated a substance with strong inhibitory activity against *Mycobacterium tuberculosis* from a strain of *Streptomyces griseus* and named it “streptomycin.” Tuberculosis has been one of the most serious infectious diseases for thousands of years, and streptomycin, as the first effective drug used in tuberculosis treatment, opened a new era of tuberculosis treatment. The discovery of streptomycin has sparked great interest among scientists worldwide in conducting antibiotic screening research. By the end of the 1940s, several antibiotics, including kanamycin, tetracycline, chloramphenicol, and erythromycin, had been successfully developed, marking the emergence of the antibiotic family (Ju-lius et al., 1980). In the 1950s and 1960s, humans ushered in the golden age of antibiotic discovery. During this period, scientists almost found that the current clinical use of the vast majority of broad-spectrum antibacterial antibiotics (cephalosporins, macrolides, glycopeptides, cyclic serine, and so on), according to statistics, these antibiotics are mainly isolated from *Streptomyces*, accounting for around 70%–80% of all isolated compounds (Bérdy, 2005); they mainly have inhibitory activity against bacteria and fungi. Tetracycline antibiotics are named after their chemical structure, which features a naphthacene core (Hertweck et al., 2007; Fritzsche et al., 2008). They are a major class of broad-spectrum antibiotics that inhibit bacterial protein synthesis, including oxytetracycline, methacycline, doxycycline, and dimethylamino tetracycline. This class of antibiotics is widely used to treat infections caused by both Gram-positive and Gram-negative bacteria, as well as intracellular pathogens such as Mycoplasma, chlamydia, and Rickettsia. Its mechanism of action involves inhibiting the binding of aminoacyl-tRNA to the ribosome, thereby suppressing bacterial protein synthesis and achieving a bacteriostatic effect (Lei et al., 2021). In addition, in some countries, including the United States, tetracycline is widely used as a growth promoter in animal feed. On the other hand, vancomycin kills bacteria by blocking the synthesis of high-molecular-weight peptide polysaccharides that comprise the bacterial cell wall, resulting in cell wall defects. Additionally, it may alter the permeability of the bacterial cell membrane and selectively inhibit RNA synthesis, thereby exerting its bactericidal effect (Qiao-zhen, 2019).

Microorganisms account for approximately 60% of the total biomass on Earth (Singh and Macdonald, 2010), making them the most diverse and abundant group of species on the planet. In the face of complex and harsh natural environments, microorganisms have evolved structurally diverse and highly active natural products (secondary metabolites) as chemical weapons, using defense, attack, or signaling to kill competitors, thereby ensuring their survival and reproduction (Seipke et al., 2012; Klassen, 2014; Traxler and Kolter, 2015). The secondary metabolites produced by microorganisms exhibit a wide range of biological activities and can serve as anticancer drugs, antiparasitic drugs, anti-inflammatory agents, herbicides, feed additives, immunosuppressants, and more, playing a crucial role in human health, pest control, and food safety. Our findings suggest that the hot spring contains a wealth of uncharacterized new clusters and a large number of unknown sub-metabolites that remain to be explored.

#### 4.2.3 Environmental potential

The rapid development of industrial and agricultural production has brought numerous conveniences to human production and life, but it has also led to the release of various pollutants into the environment. Due to their strong potential toxicity and characteristics such as bioaccumulation and biomagnification, the extensive use of environmental xenobiotics poses a serious threat to the global ecological environment and human health (Qing-ren et al., 2002). The treatment of xenobiotic pollution is urgent, and the primary methods for its remediation include physical, chemical, and biological methods. Bioremediation is the process of using the metabolic activities of specific functional microorganisms under suitable environmental conditions to reduce the activity of harmful substances in the soil or degrade them into harmless substances. Bioremediation is one of the most promising remediation methods, characterized by safety, cost-effectiveness, and minimal secondary pollution.

In recent decades, researchers have carried out a large number of fruitful studies on the chemotaxis of bacteria to environmental pollutants (Adadevoh et al., 2015, 2018) and found that the biodegradation efficiency of pollutants is not only related to the degradation ability of the degraded bacteria themselves but also depends on the bioavailability of pollutants. The bioavailability of soil pollutants is influenced by the soil media, the properties of the pollutants, and the mobility of soil microbes (Qi-shi et al., 2004). The majority of mobile bacteria can sense and search for pollutants (Lacal et al., 2013) through the chemotaxis process, and bacteria with motility and degradation abilities actively move to the adsorbed state of pollutants, which can improve pollutant bioavailability and biodegradation efficiency (Krell et al., 2013; Parales et al., 2015). The majority of organic pollutants in the environment can be degraded by microorganisms (Pieper and Reineke, 2000). Bacteria exhibit chemotaxis toward many compounds, with chemotactic substances mainly including naphthalene (Grimm and Harwood, 1997), toluene (Lacal et al., 2011), biphenyl (Gordillo et al., 2007; Tremaroli et al., 2011), polychlorinated biphenyls (Gordillo et al., 2007), benzoic acid (Gordillo et al., 2007), chlorobenzoic acid (Tremaroli et al., 2010), nitroaromatic compounds (Samanta et al., 2000), methyl parathion (Wen et al., 2007), atrazine (Liu and Parales, 2009), 2,4-D (Hawkins and Harwood, 2002), furofuran compounds (Nichols et al., 2012), and others.

Polycyclic aromatic hydrocarbons (PAHs) are organic pollutants that typically contain two or more fused benzene rings (Manousi and Zachariadis, 2020). They are highly toxic and semi-volatile (Zhao-xue and Xue-hua, 2018; Kun, 2023) and prone to long-term persistence and bioaccumulation. PAHs can migrate over long distances through various media—such as organisms, the atmosphere, water, and soil are widely distributed in the environment, making them difficult to degrade. In recent years, the pollution of PAHs in the atmosphere has become increasingly severe. The PAHs in the environment primarily originate from the incomplete combustion of oil, coal, wood, and urban waste. Additionally, the production process of petrochemical products, oil spills, leaks during oil development and transportation, volcanic eruptions, and vehicle exhaust emissions all generate PAHs. After entering the soil, it is easy to interact with soil particles and remain on their surface for a long time, making it difficult to degrade under natural conditions. Therefore, treating and repairing contaminated soil with polycyclic aromatic hydrocarbons is crucial.

Additionally, PAHs can enter the human body through the respiratory tract, digestive tract, and skin contact, exhibiting carcinogenic and mutagenic properties. Excessive exposure to PAHs often leads to lung cancer, which has the highest cancer mortality rate in the United States (Moorthy et al., 2015). Due to its severe impact on the ecological environment and human health, it has been included in the priority pollutant lists of the EU and the US Environmental Protection Agency. Although polycyclic aromatic hydrocarbon emissions are now regulated, they continue to have a significant impact on human health, so their various effects must be closely monitored.

Microbes are the most important decomposers in the ecosystem, and microbial degradation is the primary method for removing PAHs in the natural environment. Bacteria are one of the main groups of organisms belonging to the domain bacteria and are the most numerous and widely distributed type of microorganisms among all living organisms on Earth. Bacteria are widely used in the treatment of oily sludge, *in situ* remediation of oil-contaminated land, *ex-situ* remediation of oil-contaminated land, and the degradation of polycyclic aromatic hydrocarbons (PAHs) in oily sludge. Currently, it is known that many bacteria, such as *Pseudomonas, Rhodococcus, Mycobacterium, Alcaligenes, Acinetobacter* genus, *Bacillus*, and *Micrococcus*, have significant roles in the degradation of PAHs (Juhasz et al., 2010; Zhang et al., 2021). The degradation rate and efficiency of PAHs can be significantly improved by optimizing the conditions for microbial degradation, including temperature, pH, nutrient availability, and the selection of suitable microbial populations. Atrazine has become one of the most widely used herbicides due to its high efficiency, low toxicity, low cost, and broad range of applications. It is easily soluble in organic solvents such as methanol and acetone and has moderate persistence in the environment, with a half-life of 4–57 weeks in soil (Mandelbaum et al., 1993) and a half-life of up to 35 weeks in water environments (Bayati et al., 2020). The application of atrazine can lead to its spread beyond adjacent areas, contaminating soil, surface water, groundwater, and the atmosphere. Atrazine and its metabolites have been detected in various environmental media, including soil (Jablonowski et al., 2008), the atmosphere (Degrendele et al., 2022), and water (Dun-yu et al., 2022; Pan et al., 2023). Atrazine, as an endocrine disruptor, can have adverse effects on ecosystems and human health when used. In aquatic ecosystems, atrazine can alter biota and disrupt the food chains of many species, including benthic organisms (de Albuquerque et al., 2020; Xue-han et al., 2021). Degrendele and others (Degrendele et al., 2022) assessed the risk of human exposure to 30 pesticides, including atrazine. They found that infants' intake of pesticides is significantly higher than that of adults, emphasizing that infants are a vulnerable group to pesticide exposure.

To remediate environments contaminated by atrazine, researchers from various countries are dedicated to studying microorganisms that can efficiently degrade atrazine. Currently, microorganisms are isolated and have the ability to degrade atrazine, including bacteria and fungi. Bacteria play an important role in the degradation of atrazine. It has been reported that 14 genera of bacteria capable of degrading atrazine have been isolated from the environment, including *Arthrobacter* sp. (Wang and Xie, 2012), *Citricoccus* sp. (Yang et al., 2018), *Acinetobacter* (Tao et al., 2020), *Agrobacterium* (Liu et al., 2023), *Shewanella* (Ye et al., 2016), *Pseudomonas* (Fernandes et al., 2018), *Achromobacter* (Fernandes et al., 2018), *Enterobacter* (Dan-dan et al., 2017), *Rhodococcus* (Vancov et al., 2005), *Paenarthrobacter* (Zhao et al., 2025), *Klebsiella* (Zhang et al., 2019), *Pseudaminobacter* (Topp, 2001), *Micrococcus* (Sheng-wen et al., 2007), and *Streptomyces* (Mesquini et al., 2015). Five species of atrazine-degrading bacteria have been identified, namely *Agrobacterium rhizogenes* (Liu et al., 2023), *Rhodococcus erythropolis* (Vancov et al., 2005), *Paenarthrobacter ureafaciens* (Zhao et al., 2025), *Klebsiella variicola* (Zhang et al., 2019), and *Micrococcus luteus* (Topp, 2001).

Of the microbially active metabolites used today, 70% are obtained from *Actinobacteria*, the remaining 20% from fungi, 7% from *Bacillus*, and 1% from *Pseudomonas*. *Bacilli* and *Fusobacteria* are the primary components of *Firmicutes*, and the species contained within the *Bacilli* have have proven their significant application value in various fields, including agriculture, industry, environmental protection, health, medicine, and many other sectors. The thermophilic microbes from hot springs have been reported to degrade xenobiotic compounds in soil (Rong-ping et al., 2008), degrade crude oil (Shu-qian et al., 2006), fix air nitrogen (Zhang and Li, 2001), prevent plant pests (Chen et al., 2003), treat industrial wastewater, and so on. Additionally, microbes from hot springs are rich in vitamins, pigments, manganese compounds, polyunsaturated fatty acids, phosphorus, phenolic compounds, and numerous other compounds. They are a potential new type of bioactive compound “production plant” (Khalifa et al., 2021). *Cyanobacteria* exhibit significant metabolic multifunctionality, particularly in producing a wide range of structurally and functionally diverse metabolites with diverse biological activities, including antimicrobial, antifungal, antiviral, and antitumor properties. Recent research has detected a variety of compounds with anti-infectious and anti-cancer activities isolated from *Cyanobacteria* (Dixon et al., 2004). The multi-active substances produced by *Cyanobacteria* have great prospects for antimicrobial, insecticidal, antiviral, and anticancer applications (Singh et al., 2011).

The study of hot spring microorganisms not only helps us understand microbial genetic and functional diversity in high-temperature environments and their strategies for adapting to hostile habitats but also holds significant importance for screening strains with unique properties that have broad application prospects in industry, molecular biology, medicine, and other fields. This study found that the microbial resources in the hot springs of Guizhou Province are rich in species and have significant functional characteristics. There may be a substantial number of bioactive strains and related secondary metabolites with considerable biotechnological and industrial applications.

## 5 Conclusion

This study investigated the microbial diversity and industrial potential of microbial communities in 11 hot springs located in Guizhou Province, China. To our knowledge, this is the first microbiological exploration of these geothermal sites. All hot springs were rich in microbial abundance, with Guizhou Baili Rhododendron Hot Spring No. 1 being the most diverse and Jianhe Hot Spring (YAS) being the least. The phylum *Pseudomonadota* was predominant in the majority of hot springs, accounting for 41.24–92.09% of the bacterial phyla. At the genus level, *Meiothermus, Rhodococcus, Acinetobacter, Curvibacter, Rahnella, Pseudomonas*, and *Enterococcus* were among the most dominant bacterial genera in the various hot springs. Functional prediction revealed the presence of diverse metabolic categories, such as carbohydrate metabolism, amino acid metabolism, signaling, and biosynthesis of secondary metabolites. These functions aid microbial survival in harsh conditions and highlight their potential applications across various industries, including paper and pulp, tannery, textiles, food and juice, pharmaceuticals, and environmental bioremediation through the degradation of xenobiotic and toxic compounds. However, further in-depth studies using metagenomics and next-generation culturomics, combined with multidisciplinary approaches, are essential to fully harness the biotechnological potential of microbes inhabiting these hot springs.

## 6 Study limitations

This study has certain limitations. The lack of geochemical, functional metagenomic, and culturomics data hindered our ability to interpret ecological processes, microbial adaptations, and the role of microorganisms in nutrient cycling comprehensively, due to the limited number of samples and the time available. Similarly, the samples were collected at a single time under a single environmental condition.

## Data Availability

The amplicon data is submitted to NCBI as SRA under the accession number PRJNA1196608 (https://www.ncbi.nlm.nih.gov/sra/PRJNA1196608).
